# Chloroform and Polemics

**Published:** 1898-03-12

**Authors:** 


### CHLOROFORM AND POLEMICS.

The sedative effects of chloroform are no doubt great when used as a medicine, but as a topic for disputation it is among the most exciting, and is curiously provocative of angry passions and hard sayings. There is no doubt that Mr. Leonard Hill, in his papers on the subject, attacked the proceedings of the Hyderabad Commission in no measured terms. It is, however, with a certain regret that we find Dr. Lauder Brunton taking upon himself as a personal attack all that was said in regard to this investigation. Dr. Brunton states that in a lecture on the "Causation of Chloroform Syncope," delivered last year, Mr. Hill brought against the Hyderabad Commission charges of prejudice, carelessness, ignorance, and incompetence; and proceeds to say, "Although nominally directed against the Hyderabad Commission, all these charges, with the exception of the first, are really directed against me personally," and, after a long refutation of Mr. Hill's assertions, he continues, "I cannot think that Mr. Hill would publish a falsehood wilfully; but a falsehood his statement certainly is, and I shall be curious to learn how he has been led to make it." This, of course, must bring an answer from Mr. Hill, and we are afraid it means war and no quarter. It seems a pity that such a subject should give rise to such acerbities. It is, however, somewhat amusing to find that it is now nearly a year since Mr. Hill's lecture was delivered. At this rate, however ponderous the blows, the fight threatens to be slow, and will probably last our time!

